# The life and times of Professor K. N. Udupa: An outstanding alumnus of Banaras Hindu University

**DOI:** 10.4103/0975-9476.74088

**Published:** 2010

**Authors:** Ram Harsh Singh

**Affiliations:** *Department of Kayachikitsa, IMS, Banaras Hindu University, India*


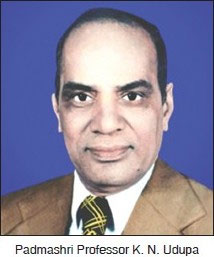
Banaras Hindu University (BHU) is one of the largest educational centers in this part of the world. It was established against all odds by Mahamana Pandit Madan Mohan Malaviya at the beginning of the last century. After a decade of preparations, the university’s foundation stone was laid on Basant Panchimi day, 1916. The vision behind it was unique, amazingly ambitious, and futuristic. It planned to focus on holistic human resource development for new nation building, adopting a strategy featuring a unique blend of tradition, cultural synthesis, and science. Although the credit of establishing the university goes largely to the Mahamana, large numbers of contemporaries, colleagues, and generous donors played commendable roles in establishing the university in its present form.

In the latter half of the 20th century, several academic members including the galaxy of over two dozen successive, highly able Vice-Chancellors contributed new ideas according to their vision. The few who contributed most to the university’s development in recent years mostly remain unnoticed. All deserve special mention for their outstanding contributions in raising the university to its present status, but among them, Padmashri K.N. Udupa figures at the top. An alumnus of BHU, he made the largest contribution to the university’s development in the post-Malavian era. His outstanding work, and more than that, his human qualities and futuristic vision, led to his being addressed as a second Malaviya on a number of occasions. This brief account touches on aspects of Udupa’s life and work in the hope that the coming generation will receive inspiration from him.

## Udupa of Kodettur from Katil to Kashi

Katil Narasimha Udupa descended from an orthodox Brahmin family of Madhawacharyas who, for a long time, served the temple of Udupi. Udupa’s grandfather, Sri Rama Krishna, migrated from Udupi to Athur to serve another temple, and later to Kodettur where a new temple that he was entrusted to serve was being constructed. Udupa’s father, Sri Tammaya Udupa, was born in 1870, as one of three sons of Sri Rama Krishna. He himself was one of six sons and two daughters of Sri Tammaya Udupa. As a scholar of Sanskrit and Jyotish, his father wanted all his sons to adopt the same profession.

Young Katil Narsimha Udupa revolted, however, opting for a medical career against his parents’ wishes. He attempted to gain admission to the Integrated Ayurvedic Medical College of Madras, but did not succeed. However, a well-wisher, impressed by his talents and keen aptitude for a medical career, advised him to try BHU in Banaras where an integrated Ayurvedic College with a good reputation already existed. He also introduced him by letter to one Prof. Dasannacharya who was then a Professor of Physics at BHU. He also told Dasannacharya that Narasimha was coming to BHU against the wishes of his parents and that there was nobody to support him financially.

In 1936, the young Udupa came to Varanasi without informing his father, Dasannacharya kept him in his family and arranged his admission to BHU’s Ayurvedic college. The young Udupa was very happy to become a student of such a reputed seat of learning. He also liked the city of Kashi, which, like his native Udupi, was a city of pilgrimage. His full interest soon made him a favorite of his teachers and, after completing six years at BHU, he passed his final AMS examination in 1943.

## POST GRADUATION

During these years, Udupa realized that his training at BHU’s Ayurvedic college would not be enough for him to become a competent doctor. This aspiration impelled him to start looking for opportunities for further study soon after graduating. he moved from Varanasi to Mumbai, and, after additional training, he proceeded to the USA where he completed his M.S. at the University of Michigan in 1948 under the patronage of Professor of Surgery, John Alexander, to whom he had been introduced by his mentor, Colonel Mirajkar, a noted surgeon in Lahore. Later, during the same trip, he completed his F.R.C.S in Canada.

Returning to India after independence in 1949, he married Nurse Lila, whom he had met in Mumbai before going abroad. In 1952, they were blessed with an only child, Anjali. Because of his Ayurvedic background, and in spite of his MS and FRCS degrees, Udupa was denied an appropriate job, but in view of his very special caliber advanced surgical training abroad, he was appointed a surgical specialist and Civil Surgeon in the Mandi District of Himachal Pradesh, where no other surgeon wanted to work. Excellent performance led to his being offered a better posting in Simla.

Declining this, he returned to the USA from 1954 to 1956 to work with J. Englebert Dunphy, the renowned surgeon and medical scientist at the Harvard University School of Medicine in Boston. Here he conducted research on wound healing mechanisms, publishing half a dozen original scientific research papers in prestigious medical journals in the USA and UK: *Annals of Surgery, New England Journal of Medicine, SGO, the British Journal of Bone and Joint Surgery,* etc. Acclaimed the world over, his work on wound healing threw new light on processes involved in wound healing and tissue repair, crucial to the science of surgical practice, and is quoted in many text books.

## VISION, MISSION AND POWER OF ACTION

In all this adventure, Udupa’s only help, besides his own talents, was his strong will and determination, and the benevolent hands of several who were impressed by his talents and sincerity. These included Dasannacharya at BHU, Mirajkar in Lahore, John Alexander of Michigan State, Harvard University’s J. Englevert Dunphy, and India’s Union Health Minister, Sushila Nayar.

Udupa returned to India again in 1956, taking up the post of Civil Surgeon in Simla, which he had earlier declined. Benevolence, dedicated services to the people, and surgical competence made him very popular among the hill peoples of Himachal Pradesh. The poor throughout the Himalayan state virtually worshipped him as a second God. His reputation soon reached higher circles in central government, and he was asked to work on policy making missions in the Government’s Ministry of Health and Family Planning.

## THE UDUPA COMMITTEE AND ITS AFTERMATH

In July 1958, he was appointed Chairman of the Committee on the Reform of Education, Practice and Research in Indigenous Systems of Medicine, the famous Udupa Committee. Vaidya Kaladi Parameshwaram Pillai of Trivandrum was another member, while an officer of the Ministry of Health, Mr. R. Narsimhan, was member-secretary. As committee chairman, Udupa led reforms of Indian Systems of Medicine (ISM), including changes in education and research at the national level. On this assignment he traveled all over the country, surveying the prevailing status of indigenous systems of medicine, so as to make a realistic report. Epoch-making recommendations for ISM’s promotion to the national level resulted. The Udupa Committee Report was submitted in April 1959 and accepted by the government, proving a milestone in the revival, development, and mainstreaming of the ISM.

The Udupa Committee Report envisaged replacing the ABMS degree by the MBBS, simultaneously starting Ayurveda post-graduate education with an M.D.Ay degree. This was based on the thinking that Ayurveda needed more research and revival efforts, which would not be possible at a routine undergraduate college, so it was initially considered for his alma mater, BHU. Udupa was of the view that, with undergraduate education already being conducted at more than 100 Ayurvedic colleges spread all over the country, BHU, as a leading center of higher education, should involve itself in higher education and research in Ayurveda and other branches of medicine. He also considered that Ayurveda would never research and development would not occur without collaboration with modern medicine and biomedical sciences. This idea was in conformity with Mahamana Malaviya’s vision in 1927, when he started BHU’s Ayurvedic College on an *integrated*pattern.

These aspects resulted in events leading to Udupa’s appointment at BHU, which indicate how famous his chairmanship of the Udupa committee had made him. Its report led BHU students to demand his posting as regular Principal of its Ayurvedic college, and they conducted several month’s strike and hunger strike to promote their demand. The resulting situation on campus forced the Prime Minister, Jawahar Lal Nehru, to intervene, and he directed Udupa to join BHU immediately in the public interest. Nehru’s decision was warmly received by all the students and staff at the university, as well as the public at large around Varanasi, and the student strike was called off. An internationally reputed surgeon had joined BHU hospital. The hope that medical services would improve came true in a very short time. Thus came about Udupa’s return to BHU in June 1959 as Professor of Surgery and Principal of the Ayurvedic College, an unwilling return, since he had no desire to serve BHU at that time.

## BANARAS HINDU UNIVERSITY

Without wasting time, Dr. Udupa quickly made drastic changes in the infrastructure of the college, including courses of study according to his vision. The university administration fully cooperated with him, granting him all the freedom required to do so. The college was converted into the “College of Medical Sciences” with the mandate to start offering the new MBBS course in modern medicine, and a postgraduate course in Ayurveda, leading to an M.D. Ay degree in Ayurveda. The MBBS course began the following year, in 1960, but the M.D. Ay degree could only begin in 1963, replacing the integrated ABMS course. The new institution combined the two systems of medicine under one roof.

The College of Medical Sciences was soon recognized by the Medical Council of India as well by the British Medical Council. This recognition brought a new status to the medical college, which until then had run as an Ayurvedic College. Only Udupa’s charismatic personality and strength of his vision could have made this possible. These qualities together with his sincerity, missionary zeal, and ability to materialize his dreams were tangibly visible, and always present to come to his aid.

This led to the development of the first big medical and health care center of its kind, catering to all the health needs of the people of the region. For this, Dr. Udupa is worshipped by the people at large, even today. He was single handedly responsible for developing BHU’s most important component, without which the university would have remained an incomplete organization at the national level. That is why many consider Udupa a second Malaviya.

The efforts of this lonely crusader and visionary activist did not stop. The infrastructure was enlarged to accommodate dozens of new courses including Super Specialty programs in both faculties. BHU’s College of Medical Sciences soon became well-known on the medical map of India. This was due both to its educational and service programs and, more, to its unique integrated character with two faculties, Ayurveda and modern medicine, under the same roof, *and* the common control and leadership of Udupa’s unique personality.

In 1972, due solely to Udupa’s efforts, hard work and selfless lobbying, the ‘College of Medical Sciences’ was upgraded to become the ‘Institute of Medical Sciences’, the short time being a record achievement. Udupa became the new institute’s Founder-Director. Among his achievements was a separate Central Surgical Research Laboratory with good modern biomedical research facilities to cater equally for the needs of researchers from both modern medicine and Ayurveda. The laboratory was used by Udupa for his own research, and by his Ayurveda research scholars for their doctoral research. It remained an active medical research center known all over the country for a number of years. Udupa would spend several hours there every day despite his extremely busy schedule as Institute Director. (The author himself was one of Udupa’s students, conducting all his PhD research in the laboratory, and, as his own Department had no facilities, for many years subsequently with his own students.)

## UDUPA THE MAN

Udupa was an excellent clinician, and a skilled surgeon whose scientific temper and human touch made him greatly loved by patients and professional associates alike. Even with BHU hospital’s meager facilities, he performed all major surgeries including mitral valve surgery on the heart, traumatic surgery on the brain, and kidney transplants – including India’s first kidney transplant in 1968. His student, A.P. Pandey, later Professor of Surgery-Urology at CMC, Vellore, subsequently conducted large numbers of kidney transplants.

Udupa made outstanding scientific contributions in the fields of his academic interest: half a dozen books, monographs, and 200+ scientific research papers in reputed, peer-reviewed, national and international journals. Study of wound healing and tissue repair was his first love, followed by a range of topics in applied and operational biomedical research. Over 50 Ph.Ds. graduated under his direct supervision and guidance, mostly in interdisciplinary areas of biomedical research. He was a remarkable research guide in that he granted his students full freedom to plan and execute their research, always encouraging an independent work culture among them. I was privileged to complete my own Ph.D. under his guidance between 1966 and 1969. As such, I owe all my ability to his teaching and guidance.

In addition to his work raising BHU’s medical campus, Udupa also contributed substantially to the management of the university as a whole. Because of his honesty, sincerity, benevolent life style, and well-known managerial abilities, he was appointed Rector and Acting Vice Chancellor of Banaras Hindu University twice, in 1967 and 1981. For lengthy periods, he tried to institute a humanistic, value-based administration in this large nationally reputed organization.

## RETIREMENT

On his superannuation on July 28, 1980, Udupa was appointed life-long Professor Emeritus. He had served BHU for 20 years, always as Principal / Director of its Institute of Medical Sciences, and he continued to serve his alma mater till his demise. For some years, he worked as a member of a joint ICMR, ICSSR panel to produce a National Report “Health for All – an Alternate Strategy.” He also rendered international service, partly as a WHO consultant, travelling widely all over the world to promote the Indian vision of health care globally. During his last years, he was engaged in a big ICMR project to develop a new, alternative model of Primary Health Care integrating Ayurveda, Yoga, and conventional medicine.

In 1992, due to extensive cancer of the colon, he underwent massive surgery at the hands of one of his finest junior colleagues, N.N. Khanna, but despite the utmost care he could not be saved. On 22 July, 1992, Katil Narasimha Udupa, the favorite of all, breathed his last after a fortnight of intensive postoperative care, in the same BHU hospital, which he himself had built and manned with so much love and dedication. It was the saddest day in the history of the institution. Thousands of his colleagues, students, and admirers from different walks of life joined his funeral at Varanasi’s Harishchandra ghat.

## SUMMARY

Udupa rose from a middle class family of orthodox Brahmins who were traditional Sanskrit scholars. His choice of a medical career was contrary to his parents’ wishes, nor was his intercaste marriage accepted by his parents. He was dedicated to his vision and self-created dreams, with amazing power of action. Repeating the conventional, beaten path was never to his liking, he was always seeking novelty. Polite but firm, sincere and truthful, honest to the core and sincere in purpose, nature provided him a handsome charming look, and a simple, charismatic personality that always arrested the attention of all with whom he came in contact. His was a unique blend of handsome personality and infectious charm, humility, and humane nature. As a visionary, he was endowed with rare courage and determination. He displayed an extreme sense of care and empathy for his patients, and great affection and encouragement to his students and colleagues. In response, he received unparallel respect and love from all corners.

In his preface to Shipra Banerjee’s biography of Udupa, N.H. Antia, the then Director of the Foundation for Research in Community Medicine, says, “*Dr Udupa’s was not a life of aggressive ambition, but a blend of remarkable humility amounting to an almost self-effacing personality. His remarkable abilities, combined with sincerity and simplicity of lifestyle, ensured love and affection from patients, students and his peers. That he was universally loved by students and colleagues alike reflects not only his personality with its remarkable honesty and integrity, but also his high professional status and respect as a surgeon, researcher and administrator.*”

Udupa was thus a legendary figure in the making of BHU, probably number one after Mahamana-ji. As well as contributing to its present state of development, he manifested qualities to inspire coming generations to follow the path of truth: sincerity, honesty, integrity, courage, commitment, and a sense of service; also the determination to translate vision and dreams into action against all odds, without undue aggressive ambition.

## SUGGESTED FURTHER READING

1. Banerjee S. Against All Odds – Story of a modern Susruta. A Biography Pub. The Foundation for research in Community Health, 84-ARG Thadani Marg, Worli, Mumbai; 1999.
2. Udupa KN. Udupa Committee Report on Indigenous Systems of Medicine, Ministry of Health Govt. of India, New Delhi; 1958
3. Udupa KN, Singh RH. Science and Philosophy of Indian Medicine. Nagpur: Baidyanath Ayurveda Bhawan; 1978
4. Udupa KN. Biology of Fracture Healing, special Research Monograph. Varanasi: BHU Press; 1966.
5. Singh RH, Udupa KN. The Kidney and its Regeneration, Research Monograph. Varanasi: BHU Press; 1974.
6. Udupa KN. Operational Research in Primary health Care. New Delhi: Indian Council of Medical Research;1991.
7. Joint Panel of ICMR and CSSR Report – Health for All, an Alternate Strategy; 1981.
8. Udupa KN. Two Decades of Medical Education in a University System: Special one time publication of Banaras Hindu University, Varanasi, India; 1980.
9. Udupa KN. Principles of General Surgery. A Text Book of Modern Surgery. Varanasi: BHU Press; 1961.
10. Udupa KN, Singh RH. Advances of Research in Indian Medicine. Special Monograph: Banaras Hindu University, Varanasi, India; 1972.
11. Udupa KN, Singh RH. Utilization of Indigenous Systems of Medicine in National Health Program – Back ground paper for the Joint Panel of ICMR and CSSR on Health for All, an Alternate Strategy; 1978.
12. Udupa KN. Disorders of Stress and their management through Yoga. Monograph, BHU, Varanasi; 1978/1985.

